# The puborectal continence reflex: a new regulatory mechanism controlling fecal continence

**DOI:** 10.1007/s00384-018-3023-9

**Published:** 2018-03-21

**Authors:** Paul M. A. Broens, Jara E. Jonker, Monika Trzpis

**Affiliations:** 1 Department of Surgery, Anorectal Physiology Laboratory, University of Groningen, University Medical Center Groningen, P.O. Box 30 001, 9700 RB Groningen, the Netherlands; 2 Department of Surgery, Division of Pediatric Surgery, University of Groningen, University Medical Center Groningen, Groningen, the Netherlands

**Keywords:** Puborectal muscle, Fecal incontinence, Fecal continence, Anorectal manometry

## Abstract

**Purpose:**

Fecal continence is maintained by voluntary and involuntary contraction of the anal sphincter, and voluntary contractions of puborectal muscle. We investigated whether the puborectal muscle can control fecal continence not only by voluntary contractions but also by involuntary contractions.

**Methods:**

We performed anorectal function tests in 23 healthy subjects. The anorectal pressure test was used to investigate voluntary contractions of the puborectal muscle. The balloon retention test was used to assess if the puborectal muscle can contract involuntarily.

**Results:**

During the balloon retention test, we observed an involuntary contraction of the puborectal muscle, which gradually increased during progressive filling of the rectum. The maximal involuntary contraction of the puborectal muscle was significantly stronger and longer than its maximal voluntary contraction (150 versus 70 mmHg, *P* < 0.001 and 5.8 versus 1.5 min, *P* < 0.001).

**Conclusions:**

We found that the puborectal muscle is able to contract involuntarily during rectal dilatation. It is a new regulatory mechanism, called the puborectal continence reflex, which controls fecal continence by involuntary contraction of the puborectal muscle. It seems to be initiated by dilatation at the level of the puborectal muscle. Presumably, the puborectal continence reflex protects many patients with anal sphincter dysfunctions from fecal incontinence.

## Introduction

Most people take fecal continence for granted. Nevertheless, approximately 0.4–15% adults and even up to 50% of elderly people living in nursing homes struggle with fecal incontinence daily; it is an enormous problem that significantly impairs their quality of life [1–3].

To date, proper diagnosis and treatment of fecal incontinence is still a challenge. Since the regulatory mechanisms of fecal continence are not completely understood, it remains an unsolved problem for many patients [4]. The classic theory of fecal continence assumes that the anus is closed by the tonic, involuntary contraction of the internal anal sphincter. In addition, voluntary contractions of the external anal sphincter and voluntary contractions of the puborectal muscle prevent fecal mass from being expelled unintentionally [5]. Previously, our research group showed that the anal-external sphincter continence reflex (AESCR) controls fecal continence by regulating involuntary contractions of the external anal sphincter [6].

Current knowledge does not completely explain the mechanisms which regulate fecal continence and which leads to fecal incontinence. For instance, although it is known that the anal sphincter plays an important role in fecal continence, some patients with severe sphincter defects remain continent or experience only a mild form of incontinence. On the other hand, there are also patients with mild sphincter defects who can be severely incontinent. It would seem, therefore, that fecal continence involves multiple regulatory mechanisms, which are not yet described.

In this study, we examined an additional mechanism for fecal continence. We hypothesize that the puborectal muscle can additionally control fecal continence by involuntary contractions.

## Methods

### Participants

We invited 50 subjects and requested them to fill in an extensive questionnaire on their defecation habits. After excluding the subjects who reported anorectal problems (*n* = 11), stress incontinence (*n* = 5), or who had a history of anorectal problems, trauma (for instance sphincter defects), or surgery in the lower gastrointestinal tract (*n* = 4), 30 healthy subjects remained. None of the women included were pregnant or had given birth prior to the study.

These 30 subjects underwent anorectal function tests in the Anorectal Physiology Laboratory at the University Medical Center Groningen. After administering the tests, we had to exclude an additional seven subjects because some of the anorectal function tests were not performed in line with the protocol (malposition of the catheter (*n* = 3), test stopped too soon (*n* = 2), malfunctioning pump (*n* = 1), balloon pressure not recorded (*n* = 1)). Finally, the measurements of 23 subjects were available for analysis.

The study was approved by the Medical Ethical Committee of the University Medical Center Groningen. The study was performed in compliance with the 1964 Declaration of Helsinki and its later amendments or comparable ethical standards. All participants gave their written informed consent.

### Measuring equipment

The anorectal function tests were performed using solar, gastrointestinal, high-resolution manometry equipment, version 8.23 (Laborie/Medical Measurements Systems, Enschede, the Netherlands), as described previously [7].

Two different anorectal function tests were performed: the anorectal pressure test and the balloon retention. Two catheters were used. Catheter 1: We used Unisensor K12981 solid-state (Boston type) circumferential catheters with an outer diameter of 12 F. This catheter measures circumferential pressure every 8 mm over a total length of 6.0 cm along the anal canal and the distal rectum. Catheter 2: We used Unisensor K14204 catheters with an outer diameter of 14 F with only two microtip sensors to connect the rectal balloon, to inflate it, and to register the pressure inside the rectal balloon.

### Anorectal function tests

We performed the anorectal pressure test to examine voluntary contractions of the puborectal muscle. During the anorectal pressure test, the subject was lying in the left lateral recumbent position and was asked to squeeze. The squeeze resulted in voluntary contractions, which were measured using catheter 1. Pressures observed during rest (i.e., basal pressure) and maximum voluntary squeeze (i.e., pressure during voluntary contractions) were used for analysis. The pressure outside the anal canal was 0 mmHg or at least less than 20 mmHg (low pressure of the buttocks).

To be sure that we were indeed analyzing voluntary contractions at the level of the puborectal muscle and not at the inner end of the anal sphincter, we defined the zone proximal of the anal canal which is localized at the level of the puborectal muscle. This was the zone where the basal pressure was lower than 15 mmHg, thus almost as low as the rectal pressure and lower than the pressure of the anal sphincter.

**Fig. 1:**
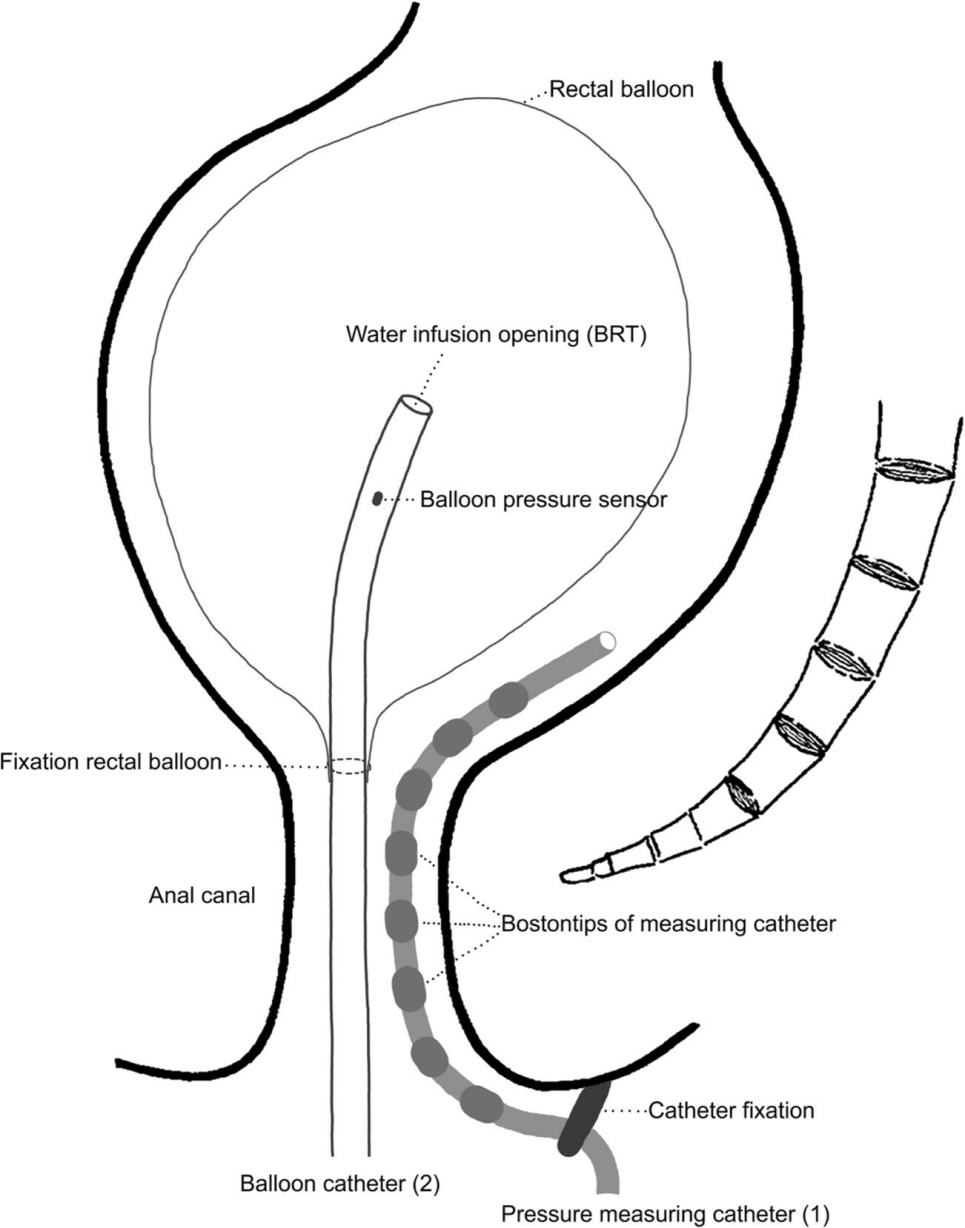
A schematic representation of the balloon retention test performed with two catheters inserted into the rectum. During the balloon retention test, the water is inflated into the balloon with catheter 2 and the measurement of the pressure is done by with fixed catheter 1

We performed the balloon retention test to examine the involuntary contraction of the puborectal muscle. During this test, the subjects were instructed to retain the balloon as long as possible. The balloon retention test was performed in sitting position. The balloon retention test is a volume-controlled system, where the rectal balloon is gradually filled (1.0 mL/s) with water of body temperature. The test allowed us to investigate if filling the rectum could evoke an involuntary contraction at the level of the puborectal muscle. The balloon retention test was stopped when the subject reached maximum tolerable sensation because none of the subjects involuntarily lost the rectal balloon. Then, the rectal balloon was immediately deflated. Thus, the balloon inflation was guided by the volume instilled and endpoint was set by the patient. We performed the balloon retention test with catheters 1 and 2 simultaneously introduced (Fig. 1). In this way, we were able to register pressures directly in the anorectum (outside of the balloon) and in the balloon. Because the compliance of the balloon itself influences the manometric results, we corrected the pressure administered within the rectal balloon for the elasticity of the balloon. Thus, the actual rectal pressure was known and used in the analysis [8]. Additionally, catheter 1 was placed outside of the balloon, along the anorectal wall, and was fixed on the buttock near to the anal canal. In this way, we minimalized the risk of movement of this catheter. The displacement of catheter 2, which is caused by continuously descending rectal content, did not influence our measurements because catheter 1 was used to measure the pressure along the anal canal and for the analysis of the contractions of puborectal muscle.

The pressure sensors were placed at 8-mm intervals on catheter 1 (Fig. 1). This way, we were able to calculate the length of “the puborectal muscle pressure zone”. The same procedure was followed in both tests.

In addition, to visualize the morphologic changes that occur during the balloon retention test, we retrospectively analyzed data collected for a previous study by Broens [9]. These data were obtained from a study on nine patients who had undergone combined anorectal manometry and proctography [9].

### Statistical analyses

The data was analyzed with SPSS 23.0 for Windows (IBM SPSS Statistics, IBM Corporation, Armonk, NY). We present values as number (percentage) or as median (range). We performed non-parametric tests as the variables were not normally distributed. We performed the Wilcoxon matched-pair signed-rank test to investigate whether there are differences between different parameters within the healthy subjects. The level of statistical significance was set at a probability of < 0.05.

## Results

### Subjects characteristic

Six male (26%) and 17 female (74%) subjects participated in our study. The mean age of the subjects was 22 (18–30) years. All subjects were able to hold the rectal balloon during the balloon retention test.

### Characteristics of voluntary contraction of the puborectal muscle

**Fig. 2:**
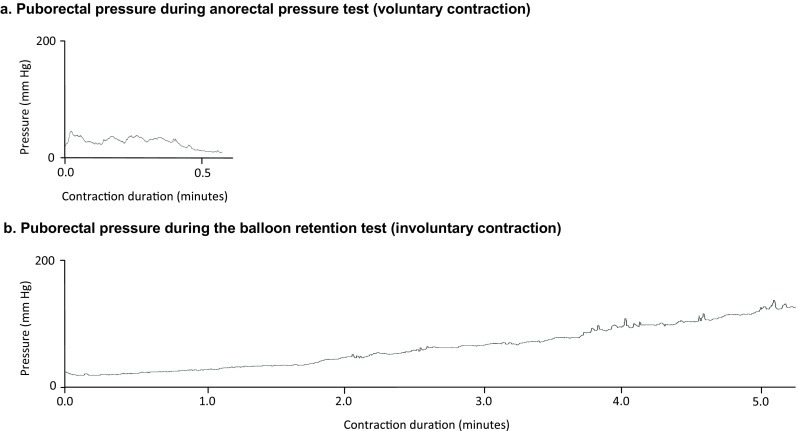
An example of voluntary and involuntary contractions of the puborectal muscle of a healthy subject. **a** Voluntary contraction: pressure gradually decreases and fluctuates. Voluntary contraction is maintained for less than 0.5 min. **b** Involuntary contractions: pressure gradually increases for at least 5 min

During the anorectal pressure test, when the subject was asked to squeeze, the pressure at the level of the puborectal muscle increased to a maximum of median 70 mmHg upon voluntary contraction. The subjects were able to squeeze with a median duration of 1.5 min, as measured by the increased pressure at the level of the puborectal muscle (Fig. 2a, Table 1). The median length of the pressure zone of the voluntary contractions at the puborectal muscle level was 1.6 cm (0.8–2.4 cm, Table 1).

**Table 1:** Voluntary versus involuntary contraction of the puborectal muscle

	Parameter measured	Voluntary contraction	*P* value	Involuntary contraction
Maximal pressure in rectal balloon (mmHg) Median (min–max)		Not applicable	Not applicable	60 (25–87)*
Maximal contraction puborectal muscle	Contraction pressure (mmHg) median (min–max)	70 (25–245)	< 0.001	150 (70–260)*
	Contraction time (min) median (min–max)	1.5 (0.5–3.0)	< 0.001	5.8 (2.7–7.8)
	Length pressure zone (cm) median (min–max)	1.6 (0.8–2.4)	< 0.05	2.4 (1.6–3.2)

*Significant difference between maximal pressure measured in the balloon compared to maximal pressure measured at the level of the puborectal muscle (*P* < 0.001)

### Characteristics of involuntary contraction of the puborectal muscle

During the balloon retention test, the subject was not squeezing, but the volume of the rectal balloon was being gradually increased instead. We observed that the pressure at the level of the puborectal muscle was also increasing due to an involuntary contraction. The pressure increased up to a maximum of 150 mmHg (Table 1). The involuntary increase appeared gradually and was observed for 5.8 min (Fig. 2b). Maximum pressure measured during involuntary contraction at the level of the puborectal muscle was significantly higher than the pressure measured in the balloon (150 versus 60 mmHg, *P* < 0.001, Table 1). The median length of the pressure zone of the involuntary contractions at the puborectal muscle level was 2.4 cm (1.6–2.4 cm, Table 1).

### Visualization of the involuntary contractions of the puborectal muscle

**Fig. 3:**
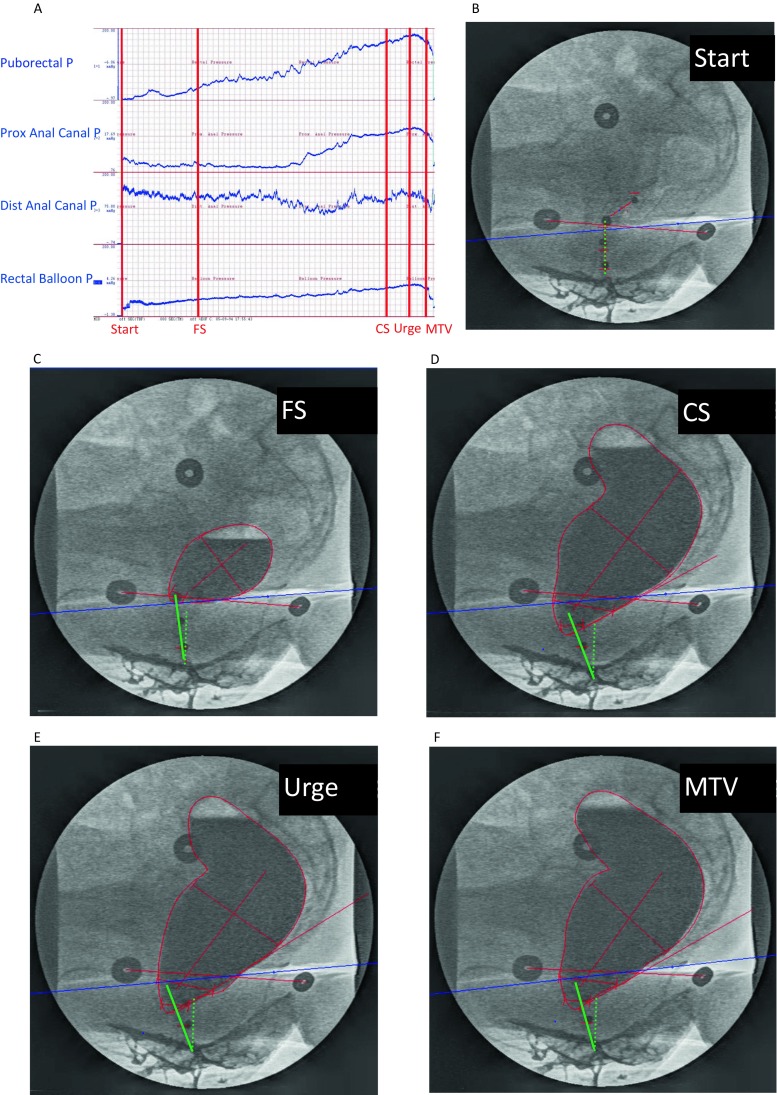
Radiologic image illustrating changes in the anorectum during gradual filling of rectal balloon in one subject. The puborectal pressure increases (**a**) during gradual filling of the rectal balloon (**a**), while the anal canal rotates in the direction of the pubic bone (**b**–**f**). The angle made by the dotted green line of anal canal at the start of the test and by the shifting line of anal canal observed during the gradual filling of the rectal balloon (continuous green line) was increasing at different stages of the measurements (**b**–**f**), i.e., at first sensation (FS), constant sensation (CS), urge sensation (US), and maximum tolerable sensation (MTS)

In addition, to actually see the anorectal response to the gradual filling of the rectum, we analyzed the outcomes of a combined anorectal manometry and proctography. We found that as the rectal balloon gradually filled, the puborectal pressure simultaneously increased from 20 (10–46) mmHg to 141 (45–221) mmHg (Fig. 3a) and the proximal part of the anal canal rotated 24° in the direction of the pubic bone from 91° (62–103) to 67° (47–92) (Fig. 3b–f). To visualize this rotation of the anal canal, we drew a dotted green line to show the position of the anal canal of one subject at the start of the balloon retention test (Fig. 3b) and a continuous green line to show the position of the anal canal at different sensation levels and at the end of this test (Fig. 3c–f).

### Voluntary contraction versus involuntary contraction

Maximum voluntary contraction was significantly lower than maximum involuntary contraction of the puborectal muscle (70 versus 150 mmHg, *P* < 0.001, Table 1). Involuntary contraction was held significantly longer than voluntary contraction of the puborectal muscle (5.8 versus 1.5 min, *P* < 0.001, Table 1 and Fig. 2).

## Discussion

In this study, we found that the puborectal muscle can contract not only voluntarily, as previously known, but also involuntarily. Muscle contractions can be described by two variables—the length of contraction and the strength as measured by changes of pressure. In this study, we focused on both parameters. We found that healthy subjects can voluntarily squeeze their puborectal muscles for approximately 1.5 min to prevent uncontrolled loss of fecal contents. The contractions we observed at the level of puborectal muscle during the balloon retention test took approximately 5.8 min (Table 1). These prolonged contractions were without any sign of gradually decreasing strength or fluctuating pressures, something we did observe during the shorter voluntary contractions (Fig. 2). This led us to conclude that the contraction of 5.8 min, during the balloon retention test, could not result from continuous voluntary squeezing of the healthy subjects who had undergone the test. Therefore, we conclude that the gradual increase of pressure, which we observed during filling of the rectal balloon, at the level of puborectal muscle resulted from involuntary contractions of the puborectal muscle. Additionally, we found that the measured involuntary contractions were significantly stronger than voluntary contractions. Furthermore, the increased pressure zone at the puborectal muscle level was significantly more extensive during its involuntary contractions than during the voluntary contractions.

Contraction of the puborectal muscle, which leads to increased angulation of the rectoanal axis, is known to support fecal continence [5]. Previously, it was thought that the puborectal muscle only contracted voluntarily, thus causing increased angulation. The same results were observed when the subjects were asked to retain the rectal balloon as long as possible while it was gradually being filled [9]. By retrospectively examining the data obtained from one of our previous studies, in which we had performed radiologic and manometric measurements simultaneously, we observed that with the increase of the pressure on the puborectal muscle, the proximal part of the anal canal rotated in the direction of the pubic bone. The combination of these two observations can only be the result of contraction of the puborectal muscle. This contraction has to be involuntary because, as we described above, it takes much longer than the duration of the maximal voluntary contraction. Previous knowledge, limited to voluntary puborectal muscle contractions, was unable to explain this “spontaneous” rotation. The current study shows that rotation of the anal canal was caused by involuntary contraction of puborectal muscle. Therefore, the increased pressure at the puborectal muscle level during the balloon retention test is considered the result of involuntary puborectal muscle contraction keeping the rectal balloon in place, i.e., providing fecal continence.

Since involuntary muscle contractions are known to be evoked by reflexes, we propose to call this mechanism, which activates involuntary contractions of the puborectal muscle, the puborectal continence reflex. Future research, to be able to properly characterize the reflex, its afferent and efferent pathways should be investigated in further studies.

### Initiation of the puborectal continence reflex

**Fig. 4:**
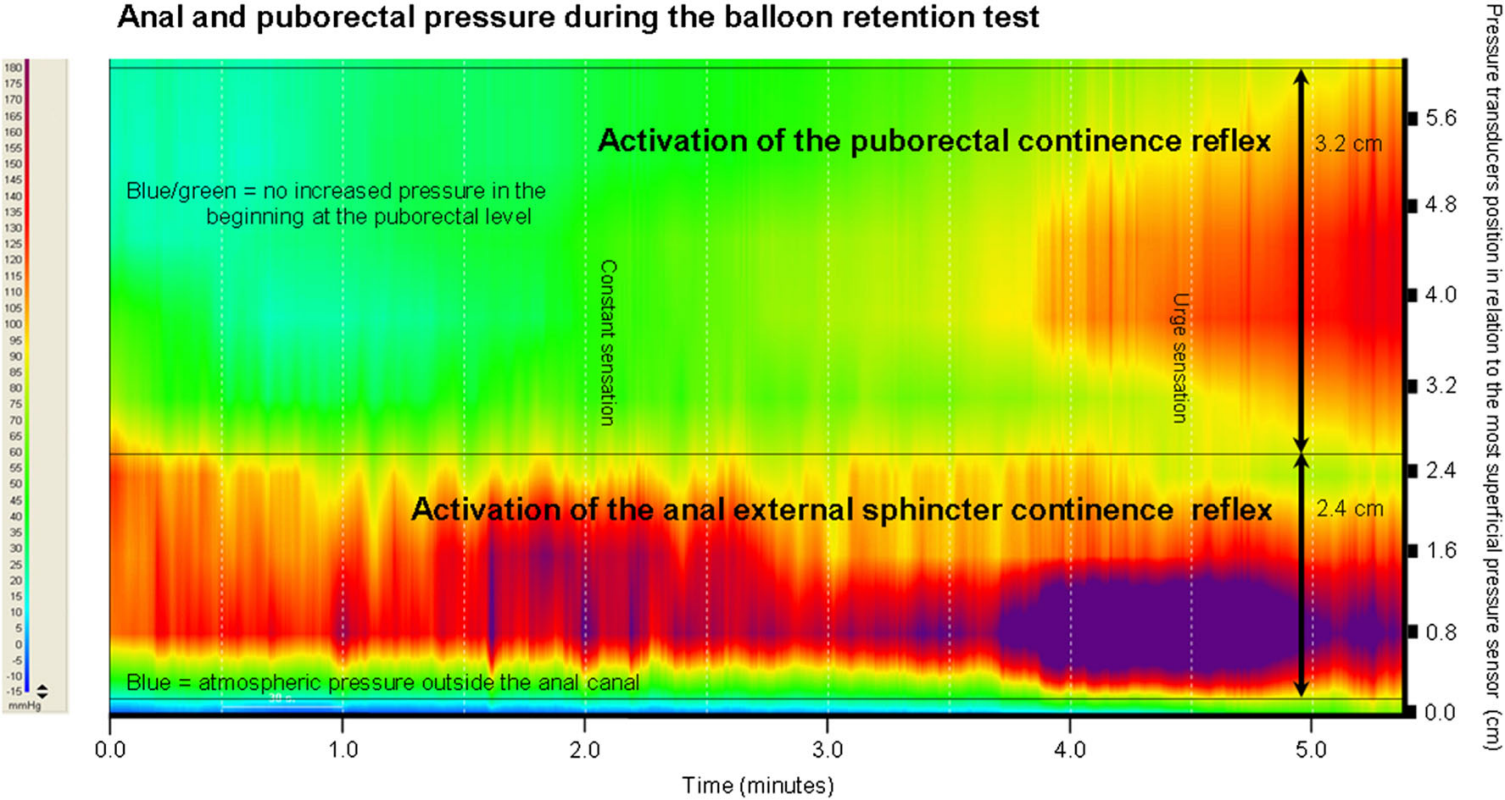
High-resolution pressure profile during gradual filling of the rectal balloon of a female subject. Puborectal and anal high-pressure zones are visible during involuntary contraction when the rectum was filled with the balloon. The color scale on the left indicates the range of anorectal pressures measured. Blue means atmospheric pressure (outside the anal canal), red/purple means high-pressure recording. The right vertical axis shows the position of the pressure transducers (black lines) in relation to the most superficial pressure sensor. The puborectal continence reflex and the anal-external sphincter continence reflex zones are indicated. The borders of the high-pressure zones are marked with black horizontal lines. Notice the activation of high pressure at the level of the puborectal muscle and the anal sphincter

In a previous study, we already described that fecal continence is controlled by the anal-external sphincter continence reflex (AESCR) that regulates the involuntary contraction of the external sphincter [6]. To illustrate the two fecal continence reflexes, the outcomes of the BRT test performed on one subject are presented in Fig. 4. In case of the AESCR, just a slight filling of the rectal balloon made the external anal sphincter contract immediately. In contrast, we observed that the puborectal muscle pressure during balloon injection was increasing gradually. Therefore, we conclude that the involuntary contraction of the puborectal muscle is activated through a different mechanism than the AESCR. We postulate that the involuntary contraction of the puborectal muscle might be activated by a stretch receptor, because gradual rectal filling resulted in gradual contraction.

### Critical evaluation

One could criticize that the pressure we measured as involuntary contraction of the puborectal muscle was actually the pressure of the balloon, thus the pressure measured in the rectum. We demonstrated, however, that the maximum pressure of the rectal balloon was significantly lower than the maximum pressure measured at the level of the puborectal muscle during involuntary contraction. Therefore, we are sure that the increased pressure we measured was the result of involuntary contraction of the puborectal muscle.

Although gender distribution is not equal, all the statistical tests performed were paired tests. Since we performed paired statistical tests, possible differences between the parameters measured in male and female subjects bear no influence on our results. Furthermore, the two groups were not compared to each other. The healthy subjects were compared to themselves, taking inter-individual variability into account, thus providing accurate analyses regardless of the differences in gender.

Although we measured the length of the pressure zone of the puborectal muscle’s involuntary contractions, it is possible that we have not yet measured the whole length of the zone. We may have missed the zone just above the catheter. However, at least, the minimum length is known. Future research is necessary to investigate the whole pressure zone.

### Clinical implications

Apparently, the puborectal continence reflex is an important continence mechanism that keeps people continent for stool by means of strong involuntary contraction of the puborectal muscle. Presumably, this mechanism prevents full fecal incontinence when the anal sphincter is not functioning well for any reason.

## Conclusion

Our results indicate that there is an additional regulatory mechanism controlling fecal continence, which involves involuntary contractions of the puborectal muscle, which we propose to call the puborectal continence reflex. The puborectal continence reflex seems to be initiated by dilatation at the level of the puborectal muscle. Presumably, the puborectal continence reflex protects many patients with serious anal sphincter dysfunctions from complete fecal incontinence.
